# Impact of Long-Term Reclaimed Water Irrigation on the Distribution of Potentially Toxic Elements in Soil: An In-Situ Experiment Study in the North China Plain

**DOI:** 10.3390/ijerph16040649

**Published:** 2019-02-22

**Authors:** Xiaomin Gu, Yong Xiao, Shiyang Yin, Honglu Liu, Baohui Men, Zhongyong Hao, Peng Qian, Huijun Yan, Qichen Hao, Yong Niu, Hui Huang, Qiuming Pei

**Affiliations:** 1 School of Geographic Science, Nantong University, Nantong 226000, China; ntugxm@ntu.edu.cn (X.G.); tempo@ntu.edu.cn (P.Q.); 2 Faculty of Geosciences and Environmental Engineering, Southwest Jiaotong University, Chengdu 611756, China; pqm@swjtu.edu.cn; 3 School of Renewable Energy, North China Electric Power University, Beijing 102206, China; mgpmbx@aliyun.com; 4 Beijing Water Science and Technology Institute, Beijing 100044, China; liuhonglu@yeah.net (H.L.); haozhongyong2002@163.com (Z.H.); 5 Geological Environmental Monitoring Central Station of Qinghai Province, Xining 810008, China; yanhj1214@aliyun.com; 6 Institute of Hydrogeology and Environmental Geology, Chinese Academy of Geological Science, Shijiazhuang 050061, China; haoqichen@mail.cgs.gov.cn; 7 Forestry College of Shangong Agricultural University, Taian 271018, China; yy_198111@163.com; 8 Department of Chemistry, Nantong Vocational University, Nantong 226007, China; ntzdhh67425@sina.com

**Keywords:** reclaimed water irrigation, potentially toxic elements pollution, soil contamination, North China Plain

## Abstract

The widespread use of reclaimed water has alleviated the water resource crisis worldwide, but long-term use of reclaimed water for irrigation, especially in agricultural countries, might threaten the soil environment and further affect groundwater quality. An in-situ experiment had been carried out in the North China Plain, which aimed to reveal the impact of long-term reclaimed water irrigation on soil properties and distribution of potentially toxic elements (As, Cd, Cr, Hg, Zn and Pb) in the soil profile as well as shallow groundwater. Four land plots were irrigated with different quantity of reclaimed water to represent 0, 13, 22 and 35 years’ irrigation duration. Pollution Load Index (PLI) values of each soil layer were calculated to further assess the pollution status of irrigated soils by potentially toxic elements (PTEs). Results showed that long-term reclaimed water irrigation caused appreciable increase of organic matter content, and might improve the soil quality. High soil organic matter concentrations conduced to high adsorption and retention capacity of the soils toward PTEs, which could reduce the risk of PTEs leaching into deep layers or shallow groundwater. Highest levels of Cr, Pb and Zn were observed at 200–240 cm and 460–500 cm horizons in plots. Longer irrigation time (35 years and 22 years) resulted in a decreasing trend of As, Cd, Hg, Pb and Zn in lower part of soil profiles (>540 cm) compared with that with 13-years’ irrigation years. Long-term reclaimed water irrigation still brought about increases in concentrations of some elements in deep soil layer although their content in soils and shallow groundwater was below the national standard. Totally speaking, proper management for reclaimed water irrigation, such as reduction of irrigation volume and rate of reclaimed water, was still needed when a very long irrigation period was performed.

## 1. Introduction

Water scarcity continues to be a major crisis in many regions in the world. An estimated 700 million people are facing water shortages in 43 countries [1,2], such as India, the State of California in the US, the North China Plain and other similar arid and semi-arid areas [3,4,5]. Overuse and extensive exploitation of groundwater have led to water shortage issues and frequent drought events [5,6], which promote reclaimed water reuse worldwide [7]. Reclaimed water is a stable water source which can contribute a huge quantity of nutrients [8,9] and its reuse in agriculture can significantly increase crop production [10]. It was estimated in a report that about 20 million ha of farmland was irrigated with reclaimed water and the amount would increase dramatically in the next few decades due to the intensive water crisis [11]. Under the circumstance, reclaimed water also carries an appreciable amount of pollution and the contents of contaminants vary from region to region depending on the source of origin [12]. In general, soil pH would be affected by the acidity of reclaimed water [13]. Electrical conductivity (EC) would increase but the soil capacity of holding nutrient elements would reduce [14]. Potentially toxic elements and organic chemicals such as polycyclic aromatic hydrocarbons were also found in the irrigated soils [10,13,15,16,17]. As a consequence, long-term reclaimed water irrigation often caused many problems with regards to progressive accumulation in soil, which may gradually destroy the soil environment and elevate the levels of contaminants in plants and shallow groundwater [18,19].

Some research had been conducted to get insight into the influence of reclaimed water irrigation on PTEs contents in soils, crops as well as groundwater [20,21,22,23]. It was found that more PTEs was accumulated in top soil layer with reclaimed water irrigation than that with clean groundwater irrigation [11,15]. Around 80% of the cumulative load of some metals, such as Cr, were retained in the top soil layer, while about 10% of the cumulative load of Ni, Zn and Cu delivered by reclaimed water was transferred to deep soil layers [24]. Therefore, many contaminants would have great possibility to transfer to deep soil layers as the reclaimed water would continue to play a major role in the agricultural production for many years. Previous studies mainly focused on the top soil layers [18,25] and paid more attention to regional content of PTEs at the horizontal scale [16,26]. Limited information is available regarding the estimation of PTEs concentrations in deep soil layer at the vertical scale, and the temporal variation of PTEs distribution in deep soil layers under different irrigation times were still poorly known. Thus it is crucial to assess the concentration of PTEs in deep soil layers under long-time irrigation to achieve better protection of soil and groundwater environment, as well as public health.

In the present study, an in-situ experiment was established to figure out the influence of long-term reclaimed water irrigation on deep soil layers as well as shallow groundwater quality. The experimental site consists of several plots which receive reclaimed water irrigation for 0, 13, 22 and 35 years, respectively. The objective of the present study is (1) to observe the physical properties of soil profiles with the depth of 620 cm under reclaimed water irrigation, (2) to estimate the influence of reclaimed water irrigation on the altered characteristics of soil including pH, electrical conductivity (EC), organic matter under different irrigation times, (3) to compare the vertical distribution of potentially toxic elements (As, Cd, Cr, Hg, Zn and Pb) under different reclaimed water irrigation times, and (4) to evaluate the soil and groundwater quality under the condition of over 30 years’ reclaimed water irrigation. This work will be significant in the rational use of reclaimed water resource and protection of soil environment and groundwater quality in long-time reclaimed water irrigation areas around the world.

## 2. Materials and Methods

### 2.1. Site Description

**Figure 1:**
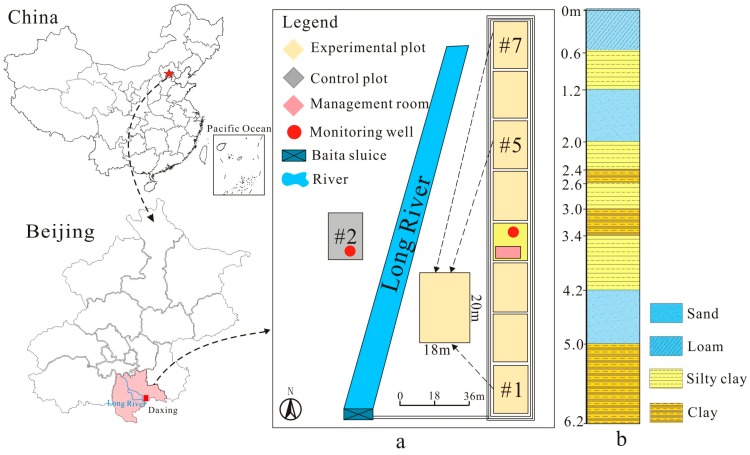
Schematic diagram of the study area. (**a**) the sampling plots from reclaimed water irrigation field at Long River reclaimed water irrigation area; (**b**) histogram of the sampling plot.

Reclaimed water irrigation began in 2002 in the North China Plain in order to alleviate the water resource crisis [20]. The Daxing reclaimed water irrigation base, located in Southern Beijing, began to use reclaimed water for agricultural irrigation in 2004. Field experimental sites in this study were located at the Long-River reclaimed water irrigation area (LRRWIA), in Daxing district (Figure 1a), which is one of the main agricultural areas in the North China Plain. The study area belongs to a semi-arid zone with continental monsoon climate. The annual mean rainfall is 504 mm, and the average evaporation per year is 979 mm [27]. Rotation of winter wheat and summer corn is the typical crop cultivation practice. The shallow aquifer groundwater depth ranges from 4 to 9 m, and that of the deep aquifer varies from 17 to 21 m. The groundwater depth in the experimental sites ranges between 2 and 14 meters [28].

### 2.2. In-Situ Experiment and Sampling

The in-situ irrigation experimental system had a serious of test plots with the area of 20 m × 18 m. Three of them (#1, #5, #7) were irrigated with reclaimed water, which was input into each plot with different irrigation rates (Table 1). The irrigation rate of plot #1 was different with plots #5 and # 7 (Table 1), which was set to figure out the influence of irrigation rate on the accumulation of potentially toxic elements in soils. The irrigation amount for the crops per year was estimated around 250 m^3^, and the conversion coefficient was set as 0.6 [29]. Simulated irrigation time of each plot was shown in Table 1, which was 13a, 22a and 35a, respectively. 

The most commonly used approach to determine the influence of reclaimed water irrigation on soil characteristics and contaminants accumulation is to compare soil properties and contaminant levels between reclaimed water-irrigated soils and non-reclaimed water-irrigated soils [10,19]. Thus, a blank control plot (#2) was set up in the western part 50 m away from the experimental plots (Figure 1a), and clean groundwater was used as the irrigation water. In order to monitor the groundwater quality under long-term reclaimed water irrigation, a monitoring well was set in the experimental site with the depth of 10 m, and the depth of the monitoring well for the blank control plot was 12 m. Shallow groundwater samples in the experimental site were collected in monitoring wells for laboratory analysis. Samples were collected in 2.5 L polyethylene plastic bottles that had been pre-cleaned with the water to be sampled. In each plot, soil samples were taken at the depth of 0–620 cm. Soil profile samples was taken every 10, 20 and 40 cm for the depth of 0–100, 100–300, and 300–620 cm, respectively. Three replications were collected per depth. For each replicate, a total of approximately 1 kg of soil was collected at four sampling points and soil subsamples at the same-depth layer were completely mixed for laboratory analysis [11,19].

### 2.3. Chemical Analysis

After removing plant roots residues, the soil samples were air-dried, ground with pestle and passed through a 2-mm sieve for analyzing soil properties (pH, EC and organic matter) and potentially toxic elements (As, Cd, Cr, Hg, Zn and Pb). Soil pH was measured using a pH meter (Orion 4 star, Thermo Scientific, Waltham, MA, USA) with a soil/water ratio of 1/2.5 for each sample. Soil EC value was determined with a benchtop conductivity meter (HI4321, Hanna, Padua, Italy). The groundwater pH and EC value were measured in situ using a multi-parameter device (Multi 350i/SET, Munich, Germany). Organic matter concentrations were measured by the oil bath heating-K_2_CrO_7_ titration method [19,31]. For soil PTEs concentration measurement, 0.5 g of ground soil samples were weighed directly into microwave PTFE vessels, with 3 mL concentrated HCl and 9 mL concentrated HNO_3_ added. The As content was determined by hydride generation-atomic fluorescence spectrometry (AFS2202, Beijing Haiguang Instrument Company, Beijing, China). The concentrations of Pb, Zn, Cr and Cd in soil and groundwater were measured using inductively coupled plasma source mass spectrometer (iCAP TQ ICP-MS, Thermo Scientific, Waltham, MA, USA), and the Hg contents were determined with cold vapor-atomic fluorescence spectroscopy (AF400CV-AFS, PG Instruments, Leicestershire, UK) [19]. The grain-size analysis of soil (clay, slit and sand) was determined by the pipette method [32].

## 3. Results and Discussion

### 3.1. Physical Analysis of Soil in Experimental Site

As the experimental plots were close to each other, the soil properties were similar as well. As a result, soil lithology was classified and merged (Figure 1b). As shown in Table 2, soil in the experimental plot was classified as silty soil based on United States Department of Agriculture (USDA) classification. 

Silt accounted for 62.2% and 81.8% at 0-0.5 m and 3–4.6 m, respectively. While the percentage of sand was relatively low, ranging from 0.7% at 0.5–1 m to 2.6% at 2–3 m, and the clay content accounted for 25.8% and 23.0% at 0–0.5 m and 4.6–6.2 m, respectively. The high content of silt and clay can reduce the vulnerability of the vadose zone as well as the risk of pollution.

### 3.2. Properties of Reclaimed Water and Groundwater for Irrigation

The physical and chemical characteristics of reclaimed water (RW) and clean groundwater (CGW) used for irrigation in this experiment are listed in Table 3. Quality Standard for Groundwater (class III) of China (GB/T14848-93) was based on the human health benchmark value, and the class III groundwater was mainly utilized for centralized drinking, industrial production and agricultural irrigation. The pH values of RW and CGW were 7.7 and 7.5, respectively, and fell within the guideline, which ranges between 6.5–8.5. The electrical conductivity (EC) was 2120 μS/cm for RW and 946 μS/cm for CGW, indicating a moderate and low level of salinity, respectively [33]. As expected, the content of almost all the trace elements in RW was higher than that in CGW, except for Pb (Table 3). Cd and Hg values of RW were over the China guideline. According to the chemical characteristics, the high content of some trace elements in the RW used for irrigation may pose PTEs pollution risks.

### 3.3. Effects of Reclaimed Water Irrigation on Soil Characteristics

Data with different irrigation times showed the influence of reclaimed water irrigation on soil chemical properties. The statistical analysis results, including pH, EC and organic matter, are shown in Table 4. 

**Figure 2:**
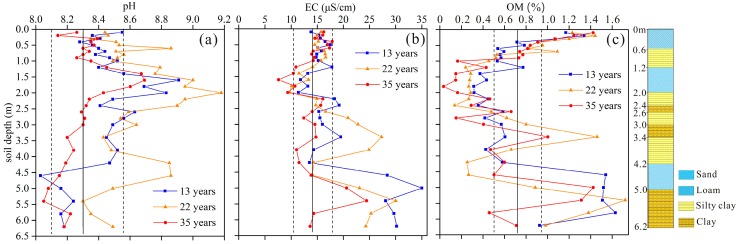
pH, EC and organic matter (OM) concentrations in soil profiles with different irrigation times. Solid line represents the mean value of control plot, and dashed lines represent corresponding standard deviations.

The mean pH values display an increasing trend at topsoil (0–200 cm) and a decreasing trend on the soil profile below 200 cm. The largest pH value existed at 100~200 cm with 22 irrigation years, which had a mean value of 8.94. Combined with Figure 2a, soil pH values have been slightly affected at top one meter with long term irrigation time and showed no significant differences (*p* > 0.05) between pH data from control plot and experimental plot. 

The mean pH value was 8.57 in the plot with no reclaimed water irrigation at a depth of 100–200 cm. The increasing pH values in the soil profile (100–200 cm) was related to the alkaline pH values of the reclaimed water (the pH value of reclaimed water sampled in April 2016 was 7.6). The anaerobic environment of the clay soil layer might result in cation displacement or the addition of weak inorganic alkali, i.e., NH_4_^+^, or excessive leaching of acid cations, which can explain the declining trend of pH below 200 cm, as well as the decreasing trend of pH values after 35 irrigation years. In the present study, reclaimed water irrigation caused an increase of pH values in the top soil layer compared to that in the control plot, while a decrease of pH values was observed in many irrigated soils where the irrigation reclaimed water was acidic [10,33].

Results of EC determination of water saturate soil extracts (Table 4 and Figure 2b) showed that long-term irrigation with reclaimed water had led to an increase in the salinity content with the increasing depth in topsoil (0–150 cm), which was related to the salts in the reclaimed irrigation water as well as a preponderance of upward water movement and evaporation at the topsoil surface [34]. The EC values with different irrigation times showed no significant differences at the depth of 0–200 cm and higher EC values were found at sandy layers (250–350 cm and 400–500 cm). The mean EC values of sandy layers with irrigation times of 13, 22 and 35 years were 22.8, 19.2 and 14.6 μS/cm, respectively, which were higher than that of the control plot (value of 13.9 μS/cm). The EC values declined with increasing irrigation time, and were related to the continual leaching of salts to lower horizons under the high irrigation rate with reclaimed water [35], and the mobility of sandy layers was higher than that of clay layers. Considering the field and crops tolerances to salinity, the salinity might not be a serious problem under the condition of adequate over-irrigation.

The OM concentrations in the soil profiles ranged between 0.035% and 1.72% from topsoil to lower layers (Table 4 and Figure 2c). Compared to the pristine concentration of reclaimed water, soil OM in the top 100 cm was increased by 9.3%, 14.9% and 32.7% with reclaimed water irrigation times of 13, 22 and 35 years, respectively. The accumulation of OM in reclaimed water irrigation field could improve soil structural properties and increase soil fertility and crop production as well [36]. The mean content of organic matter ranged from 0.19 to 0.44% at 100–200 cm. It was relatively low with a small percentage of clay and silt. In addition, soil pH values were also higher at 100–200 cm than those at other depths, and the anaerobic alkaline soil environment could explain the decrease of organic matter. As a result, a significant increase of OM was found at 260–340 cm and 460–500 cm. High soil OM concentration was reported to be able to reduce the mobility of PTEs and the risk of leaching as well [37]. Generally speaking, long-term reclaimed water irrigation would lead to appreciable increase of OM content, and might improve the soil quality.

### 3.4. Effects of Reclaimed Water Irrigation on the Accumulation of Potentially Toxic Elements

**Figure 3:**
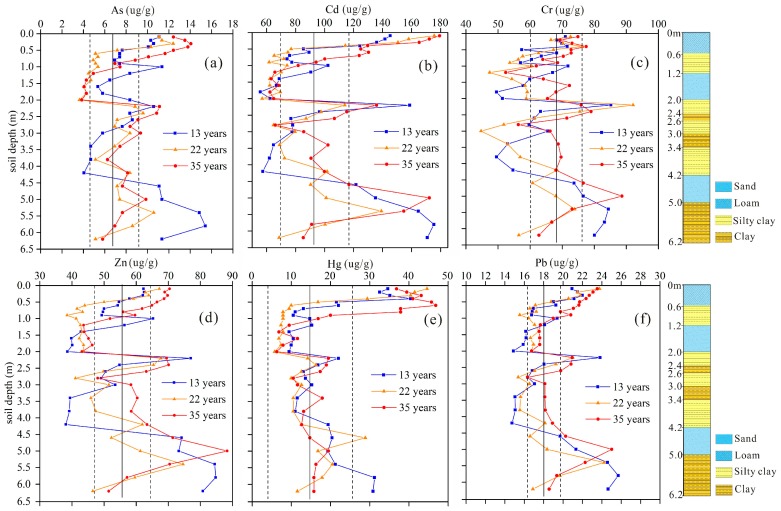
PTEs values in soil profiles with different irrigation times. Solid line represents the mean value of control plot, and dashed lines represent corresponding standard deviations.

The statistical analysis of PTEs in experimental plots as well as control plot (the one with clean groundwater irrigation) was shown in Figure 3 and Table 5. The concentrations of most elements have an increasing trend with the increase of irrigation years compared with that of the control plot. A growth in standard deviation values also indicated that anthropogenic factors have an influence of the concentration and distribution of PTEs in soil profile [21]. The largest soil As values were observed in top soil layer (0–100 cm) with mean values of 7.33–11.09 μg/g, while in the depth of 100–200 cm, the mean As contents decreased and varied from 4.06 to 6.87. This phenomenon was the result of large percentage of sand in the soil profile of 100–200 cm, most As was delivered by reclaimed water to deep soil layer. As a result, the As values displayed an increasing trend from 200 cm to 300 cm. 

The same phenomenon could be observed in the soil profile of Cd, Cr and Zn. For the six elements examined, the highest levels of Cr, Pb and Zn were observed at 200–240 cm and 460–500 cm horizons in plots (Figure 3), which was the result of the large percentage of clay and silt in the two layers. The soils with high content of clay and silt had strong adsorption capacity and facilitate the metals to accumulate in clay soil layers. However, PTEs accumulation in topsoil (0–100 cm) might enrich the concentrations of the associated crops and led to certain risks to crop growth or even human health [38]. 

Figure 3 also suggested that longer irrigation times (35 years and 22 years) led to a decreasing trend of As, Cd, Hg, Pb and Zn in the deep layers of the soil profiles (>540 cm) compared with that with 13-year’ irrigation. For instance, the mean As contents at the depth of 500–620 cm under 13, 22 and 35 years’ irrigation were 13.87, 8.10 and 6.79 μg/g, respectively (Table 5). The soil As value under 35 years’ irrigation was even lower than that of the control plot with groundwater irrigation. Considering the continual input of elements through reclaimed water irrigation and the adsorption of PTEs on soil particles, this could be the result of plant uptake and leaching through sandy soil layers. Additionally, high soil OM concentrations led to high adsorption and retention capacity of the clay and silty soils toward PTEs, which was the major component to fix PTEs in soils and could reduce the risk of PTEs leaching into lower layers or groundwater [27,30]. The mobility of PTEs was also enhanced by high loading of around 250 m^3^ reclaimed water irrigation per year, which was related to much lower concentration of PTEs under long-term irrigation conditions.

### 3.5. Evaluation of Irrigated Soils and Shallow Groundwater Quality

To further assess the pollution status of irrigated soils by PTEs, the Pollution Load Index (*PLI*) was applied to evaluate the pollution extent, which is based on the concentration factor values (*CF*) of individual PTE in the soil [39]. The *CF* value is the ratio of the content of each metal (*C_m_*) to the background value in the soil (*C_b_*):

(1) $$CF=\frac{c_{m}}{c_{b}}$$

where the contents of PTEs in control soils (#2) were chosen as background values, and PTEs concentrations (*C_m_*) used were the values of soil samples. And PLI was defined as the geometric mean value of *CF* values for the *n* metals as the following equation:

*PLI* = (*CF_1_* × *CF_2_* × … × *CF_n_*)*^1/n^* (2)

**Figure 4:**
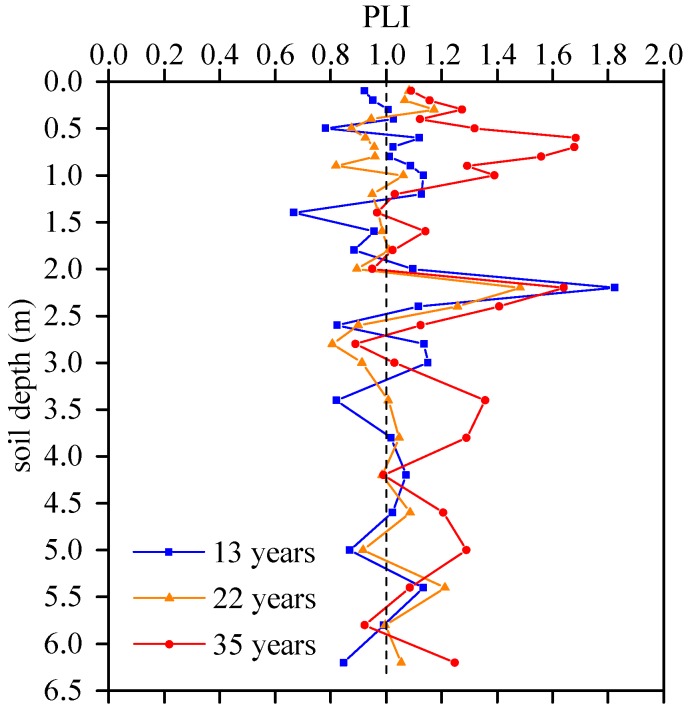
PLI values in soil profiles with different irrigation times. Dashed lines represent value of 1.

Values of PLI ≤ 1 show PTE loads close to the background level, and values over 1 indicate pollution [40]. PLI values of each soil layer were shown in Figure 4 and indicated that most PLI values ranged between 1 and 2. 

Plots with 35 years’ irrigation had the highest PLI values in topsoil (0–300 cm), which is related to large amount of reclaimed water irrigation and the adsorption of loam soil. There was a significant decrease of PLI at 60 cm for all plots, which was related to the high adsorption effect of clay and silty soil. Plots #5 and #7 had smaller PLI values at 200-230 cm than that of plot #1, which is associated with the high infiltration rate and leaching through sandy soil layers, with the progressive accumulation of PTEs, plot #7 accumulated more PTEs than that of plot #5 although they had the same infiltration rate. The PTEs concentrations exhibited a decreasing trend from 240 cm to deeper soil layers with 13 years’ irrigated time, which means the influence of reclaimed water irrigation on plot #1 is not significant and even the background values of PTEs were diluted. However, PLI value reached 1.05 and 1.24 at 620 cm for plot #5 and plot #7, respectively. The results showed that long-term reclaimed water irrigation (>30 years) had polluted deep soil environment and might have potential risk for shallow groundwater.

PTE values in groundwater under long-term reclaimed water irrigation are shown in Table 6. Groundwater Quality Standard (class I) reflects the natural low background content of groundwater chemical components and suitable for various purposes. 

Most PTEs values in groundwater fell within the guideline and similar to the background values, indicating that the PTEs in reclaimed water, including As, Cd, Zn, Hg and Pb, pose little risk to shallow groundwater quality under long-term irrigation conditions (>30 years), whereas, Cr values in shallow groundwater are higher than the guideline, which was related to the historical presence of an electroplating factory, whereby sewage discharge from the factory increased the background value of Cr in the vadose zone. Although under the condition of 35 years’ irrigation, the reclaimed water have little influence on PTEs contents in soil environment and shallow groundwater, proper management for reclaimed water irrigation, such as reduction of irrigation volume and rate of reclaimed water, is still needed when irrigation for a very long period is performed.

## 4. Conclusions

Reclaimed water used as irrigation water could provide soils with nutrients and organic matter, thus improving soil fertility and crop production. However, irrigation of agricultural fields with reclaimed water can change some soil physical and chemical properties. Generally speaking, the pH values and the salinity content in the top soil layer were increased compared to that in the control plot. Long-term reclaimed water irrigation also led to appreciable increase in the concentration of organic matter, and might improve the soil quality in top soil layer. PTEs would progressively accumulate on top soil layers (0–250 cm) under long-term reclaimed water irrigation. Due to the high adsorption and retention capacity of clay and sand-clay interlayers in the vadose zone, the PTEs were adsorbed and accumulated in these aquitard layers, while for the sandy layers, PTEs were inclined to leach when the infiltration rate is high and would accumulate if the infiltration rate was small, thus the concentrations of PTEs in deep soil layer with 35 and 22 years’ irrigation time were lower than that of 13 years’ irrigation time. Although under the condition of 35 years’ irrigation, the reclaimed water has little influence on the PTE contents in soil and shallow groundwater, excessive inputs of some elements could also have adverse impact on the deep soil layers and might cause pollution in shallow groundwater when irrigation is performed for a very long period. It is suggested that work for investigating the influence of reclaimed water irrigation on unsaturated-saturated system is necessary. Risk assessments should also be carried out in order to keep safe usage of reclaimed water for agriculture, as well as the protection of soil environment and groundwater quality.

## Figures and Tables

**Table 1:** The data of the in-situ experiment.

Plot No.	Leakage Depth ^a^ (m)	Total Leakage Amount ^b^ (m^3^)	Simulated Irrigation Time ^c^ (a)	Irrigation Rate (cm/h)
#1	6.1	2210.2	13	0.5
#5	10.4	3740.6	22	5
#7	16.5	5950.4	35	5

^a^ The leakage depth was measured in the field [30]; ^b^ The leakage amount equals the leakage depth times the leakage area (20 m × 18 m); ^c^ Simulated irrigation time is the ratio of the total leakage amount to the leakage amount per year, the latter equals the conversion coefficient times the irrigation amount for the crops per year.

**Table 2:** Soil physical properties of the experimental plot.

Depth (m)	Coarse Sand (%)	Fine Sand (%)	Very Fine Sand (%)	Silt (%)	Clay (%)
0~0.5	—	—	12.0	62.2	25.8
0.5~1	—	0.7	27.1	51.1	21.1
1~2	1.3	2.0	56.5	36.2	4.0
2~3	—	2.6	30.8	55.2	11.4
3–4.6	—	—	1.9	81.8	16.4
4.6–6.2	—	—	4.5	72.5	23.0

Coarse sand 200 < *φ* < 2000 μm; Fine sand 50 < *φ* < 200 μm; Very fine sand 20 < *φ* < 50 μm; Silt 2 < *φ* < 20 μm; Clay *φ* < 2 μm.

**Table 3:** Physical and chemical characteristics of the irrigation waters used in the plots.

Index	RW	CGW	Groundwater Quality Standard (Class III)
pH	7.7 ± 0.1	7.5 ± 0.11	6.5–8.5
EC (μS/cm)	2120 ± 2.0	946 ± 1.0	—
As (μg/L)	18.5 ± 0.01	2.0 ± 0.01	50
Cd (μg/L)	17.0 ± 0.02	5.0 ± 0.01	10
Cr (μg/L)	12.2 ± 0.02	4.0 ± 0.01	100
Zn (μg/L)	115.2 ± 0.04	6 ± 0.03	200
Hg (μg/L)	5.0 ± 0.01	0.01 ± 0.01	1.0
Pb (μg/L)	0.7 ± 0.02	0.9 ± 0.01	20

Data represents mean values ± standard deviation. EC: electrical conductivity; RW: reclaimed water; CGW: clean groundwater.

**Table 4:** Statistical analysis of soil characteristics in different soil depth with different irrigation years.

Parameters	Irrigation	Mean ± SD Values in Different Depth				
	Years	0–100 cm	100~200 cm	200~300 cm	300~500 cm	500~620 cm
pH	0	8.30 ± 0.29	8.57 ± 0.14	8.35 ± 0.10	8.27 ± 0.11	8.13 ± 0.02
	13	8.42 ± 0.08	8.68 ± 0.18	8.52 ± 0.07	8.33 ± 0.19	8.19 ± 0.03
	22	8.52 ± 0.13	8.94 ± 0.15	8.72 ± 0.17	8.62 ± 0.19	8.38 ± 0.08
	35	8.31 ± 0.07	8.57 ± 0.11	8.31 ± 0.02	8.17 ± 0.05	8.15 ± 0.07
EC(μS/cm)	0	14.62 ± 6.03	13.06 ± 0.80	15.10 ± 3.55	12.49 ± 2.54	15.10 ± 2.12
	13	15.45 ± 0.95	13.48 ± 2.28	16.86 ± 1.59	26.14 ± 13.04	29.33 ± 0.90
	22	15.55 ± 0.69	11.74 ± 1.72	17.98 ± 3.28	20.74 ± 5.50	33.20 ± 6.58
	35	15.54 ± 1.39	9.78 ± 1.23	14.50 ± 1.27	14.32 ± 3.43	17.43 ± 4.93
OM (%)	0	0.92 ± 0.32	0.27 ± 0.02	0.46 ± 0.24	0.71 ± 0.45	1.36 ± 0.30
	13	0.79 ± 0.28	0.44 ± 0.17	0.48 ± 0.09	0.93 ± 0.49	1.36 ± 0.31
	22	0.92 ± 0.32	0.27 ± 0.02	0.46 ± 0.24	0.71 ± 0.45	1.36 ± 0.30
	35	0.87 ± 0.32	0.19 ± 0.13	0.39 ± 0.17	0.80 ± 0.36	0.83 ± 0.36

**Table 5:** Statistical analysis of PTEs in different soil depth with different irrigation years.

PTEs	Irrigation Years (a)	Mean ± SD Values in Different Depth				
		0–100 cm	100~200 cm	200~300 cm	300~500 cm	500~620 cm
As (μg/g)	0	7.33 ± 2.6	4.06 ± 0.3	7.79 ± 2.1	7.0 ± 1.4	7.22 ± 1.9
	13	9.02 ± 1.7	6.87 ± 1.3	8.19 ± 1.5	7.18 ± 3.3	13.87 ± 1.8
	22	7.82 ± 2.9	4.23 ± 0.4	8.17 ± 1.0	7.00 ± 1.1	8.10 ± 2.3
	35	11.09 ± 2.5	4.27 ± 0.4	9.74 ± 1.1	7.84 ± 1.2	6.79 ± 0.8
Cd (μg/g)	0	104.5 ± 27.2	70.96 ± 7.7	89.24 ± 12.7	92.46 ± 20.6	94.8 ± 20.9
	13	105.2 ± 27.4	68.88 ± 11.8	97.52 ± 31.5	88.22 ± 33.2	170.33 ± 4.4
	22	102.3 ± 41.3	64.18 ± 4.9	88.58 ± 16.6	86.94 ± 13.9	102.73 ± 28.8
	35	129.9 ± 31.6	64.86 ± 2.1	101.8 ± 24.1	116.3 ± 29.1	110.2 ± 31.3
Cr (μg/g)	0	70.40 ± 6.6	68.72 ± 1.9	69.95 ± 12.4	63.06 ± 7.7	66.78 ± 5.3
	13	65.94 ± 5.5	57.11 ± 6.3	66.99 ± 9.3	61.49 ± 11.3	82.58 ± 1.8
	22	64.37 ± 7.0	55.59 ± 4.5	65.08 ± 17.0	61.41 ± 6.1	65.65 ± 7.2
	35	70.45 ± 4.5	64.41 ± 6.6	69.70 ± 7.9	74.25 ± 7.7	67.46 ± 4.2
Hg (μg/g)	0	22.05 ± 13.8	6.84 ± 0.3	10.58 ± 1.1	12.57 ± 2.4	13.78 ± 2.1
	13	23.57 ± 10.7	10.92 ± 2.2	16.17 ± 3.2	16.34 ± 4.2	27.79 ± 4.6
	22	21.58 ± 14.8	7.04 ± 0.7	13.13 ± 2.1	15.86 ± 6.9	16.63 ± 3.7
	35	36.49 ± 9.6	8.40 ± 1.9	15.54 ± 3.8	15.54 ± 2.7	15.93 ± 0.3
Pb (μg/g)	0	19.16 ± 1.9	17.16 ± 0.1	17.46 ± 0.4	17.05 ± 1.9	18.15 ± 0.9
	13	19.38 ± 1.9	16.25 ± 1.1	18.42 ± 2.8	17.18 ± 2.8	24.98 ± 0.5
	22	18.93 ± 2.6	16.98 ± 0.3	17.84 ± 2.0	16.85 ± 1.2	20.26 ± 3.0
	35	21.54 ± 1.4	17.41 ± 0.3	19.18 ± 1.7	20.07 ± 2.6	20.04 ± 1.6
Zn (μg/g)	0	54.83 ± 7.4	48.2 ± 1.2	58.42 ± 2.9	58.28 ± 10.3	60.97 ± 9.7
	13	56.81 ± 5.7	43.54 ± 6.6	56.98 ± 10.2	52.84 ± 17.1	83.37 ± 1.8
	22	51.79 ± 10.4	43.06 ± 0.6	55.1 ± 10.0	53.76 ± 6.7	60.23 ± 11.5
	35	63.77 ± 6.0	44.38 ± 1.2	61.76 ± 8.1	68.29 ± 10.9	59.62 ± 7.9

**Table 6:** PTEs values in shallow groundwater of monitoring wells.

Types	PTEs Values in Groundwater (ug/L)					
	As	Cd	Cr	Zn	Hg	Pb
Monitoring well (experimental site)	1.9	0.013	18.8	1.9	0.012	0.21
Monitoring well (control plot)	2.1	0.006	15.3	1.5	0.009	0.16
Groundwater Quality Standard (class I)	5.0	0.1	5.0 (Cr^6+^)	50	0.05	5.0
